# CD38 identifies pre-activated CD8+ T cells which can be reinvigorated by anti-PD-1 blockade in human lung cancer

**DOI:** 10.1007/s00262-021-02949-w

**Published:** 2021-05-02

**Authors:** Pin Wu, Lufeng Zhao, Yongyuan Chen, Zhongwei Xin, Mingjie Lin, Zhixing Hao, Xiaoke Chen, Di Chen, Dang Wu, Ying Chai

**Affiliations:** 1 Department of Thoracic Surgery, Second Affiliated Hospital, Zhejiang University School of Medicine, Zhejiang University, Hangzhou, 310009 China; 2 Key Laboratory of Tumor Microenvironment and Immune Therapy of Zhejiang Province, Second Affiliated Hospital, Zhejiang University School of Medicine, Zhejiang University, Hangzhou, 310009 China; 3 Department of Clinical Laboratory, The Second Affiliated Hospital, Zhejiang University School of Medicine, Zhejiang University, Hangzhou, 310009 China; 4 Department of Oncology Radiotherapy, Second Affiliated Hospital, Zhejiang University School of Medicine, Zhejiang University, Hangzhou, 310009 China

**Keywords:** CD8+ CD38+ T cells, Non-small cell lung cancer, Immunotherapy

## Abstract

**Background:**

CD38 has been observed expressing in activated T cells, while the features and functions of CD38+ T cells in human NSCLC are still unclear.

**Methods:**

Here we uncovered the correlation between CD38 expression and survival and immune infiltration levels in tumor of NSCLC. Then, we collected samples from 51 NSCLC patients to study the biological feature and response to anti-PD-1 of tumor-infiltrating CD38+ CD8+ T cells in vitro.

**Results:**

We found CD38 expression correlated with the survival and immune infiltration levels of NSCLC. It is interesting that CD38+ CD8+ T cells enriched in the tumors expressed higher level of cytotoxic molecule, cytokines and PD-1 than CD38− CD8+ T cells. Moreover, PD-1+ subset in tumor-infiltrating CD38+ CD8+ T cells expressed higher level of activated markers than PD-1+ CD38− CD8+ T cells. Next, we found tumor-infiltrating CD38+ CD8+ T cells expressed higher level of CD103, IFN-γ, TNF-α and perforin than CD38− CD8+ T cells when were reactivated in vitro. Finally, we observed that CD38+ CD8+ T cells isolated from tumors could be reinvigorated by anti-PD-1 in vitro.

**Conclusions:**

Our findings demonstrate that CD38 expression defines a subset of CD8+ T cells enriched in tumors of NSCLC which have paradoxical phenotypes and response to anti-PD-1. Our results suggest a pre-priming of these cells is may exist in tumor and consequentially facilitate it acquiring both anti-tumor potency and exhausted phenotype which can be reinvigorated by PD-1 blockade.

**Supplementary Information:**

The online version contains supplementary material available at 10.1007/s00262-021-02949-w.

## Introduction

Non-small-cell lung cancer (NSCLC) is a common malignant tumor causing a leading morbidity and mortality worldwide [1]. Especially in China, the incidence and fatality rate of NSCLC are on the top of the list and still show an upward trend in recent years [2]. Although there are multidisciplinary treatments, such as surgery, chemotherapy, radiotherapy, and targeted drug therapy for NSCLC, its overall survival rate is still unsatisfactory. Therefore, it is necessary to identify potential target molecules or target cells and developing new therapeutic strategies that can improve the clinical benefits of NSCLC patients.

CD38, a single chain type II transmembrane molecule displaying a canonical molecular weight of ~ 45 kDa, plays diverse roles in human cells, such as receptor, adhesion, molecule and ectoenzyme [3, 4]. Initially found on thymocytes and T lymphocytes [5], CD38 was considered as an activation molecule [3, 6], involved in lymphocyte activation, proliferation and adhesion [7, 8]. More importantly, as the indicator of functional feasibility [9], CD38 is a vital surface marker in CD8+ T cells, which can act as surface molecule by regulating the intracellular levels of calcium and downstream signaling pathways through its ADP-ribosyl cyclase activity [4]. However, there are also some studies suggesting that CD38 can act as an immune checkpoint for T cells [10]. Besides, depletion of CD38+ immune regulatory cells resulted in an increase in T-helper cells, cytotoxic T cells, T-cell functional response and TCR clonality in multiple myeloma [11]. Thus, CD38 may be a multifunctional molecule and the property of CD38 as a surface marker on CD8+ T cells has not been confirmed. Therefore, to identify the functional characteristics of CD38+ CD8+ T cells in NSCLC is demanding.

Anti-PD-1 blockade is an emerging treatment method in recent years, which mainly reinvigorates the original functions of immune cells by blocking immune check points on their surface, thereby eliminating foreign pathogens or tumors [12]. Unfortunately, although anti-PD-1 has revolutionized the treatment for several NSCLC subtypes in recent years [13, 14], key mechanisms determining populations suitable for immunotherapy or subgroups of immune cells determining anti-tumor efficacy in NSCLC remain largely unknown. With the crucial capability of directly killing tumor cells, tumor-infiltrating CD8+ T cells have been suggested to be effectively activated by anti-PD-1 blockade, and consequently dominate immunotherapy mechanism in NSCLC [15]. However, it is still unclear which subgroups of CD8+ T cells play such vital roles in immunotherapy. Previous studies have identified several subgroups of CD8+ T cells responsible of anti-tumor effect in tumor-killing process [15, 16], which implicated our current exploration on whether the subgroup of CD38+ CD8+ T cells also act as a member involved in tumor-killing process, in the hope of helping improve the success rate of immunotherapy for lung cancer patients [17].

To explore the role of tumor-infiltrating CD38+ CD8+ T cells in NSCLC and whether they can be used as target cells for immunotherapy, we investigated the prognostic value, functional characteristics and response to anti-PD-1 antibody of CD38+ CD8+ T cell subsets in vitro. Here, we found that increased expression of CD38 was associated with better clinical outcomes of NSCLC patients. Moreover, tumor-infiltrating CD38+ CD8+ T cells showed a pre-activated phenotype and expressed higher level of PD-1. It is interesting that tumor-infiltrating CD38+ CD8+ T cells are more efficient to be reinvigorated by anti-PD-1 than CD38− CD8+ T cells in vitro. Our study suggest that increased CD38 expression defines tumor-infiltrating CD8+ T cells been pre-activated which involved in anti-tumor immunity through secreting cytotoxic molecule and also may function as the target cells of anti-PD-1 blockade.

## Materials and methods

### TIMER database

Tumor Immune Estimation Resource (TIMER, cistrome.shinyapps.io/timer) is a new website that involves 10,897 samples across 39 cancer types from The Cancer Genome Atlas (TCGA) for estimating the level of immune infiltrates and it provides six major analytic modules to deeply excavate molecular characterization of tumor-immune interactions including Gene module, Survival module, Mutation module, SCNA module, Diff Exp module and Correlation module. We first analyzed CD38 expression in different types of cancer by using Diff Exp module. And then, we explore the clinical relevance of LUAD and LUSC via Survival module. Next, we find out the correlation between the level of CD38 expression and immune infiltrates, including B cells, CD4^+^ T cells, CD8^+^ T cells, neutrophils, macrophages and dendritic cells, via gene modules in diverse cancer types. The gene expression level converts into log2 RSEM.

### Tissue collection

Tumor (T, homogeneous cellularity, without foci of necrosis), paired normal lung tissues (N) and some fresh peripheral blood (PB) were obtained from patients with NSCLC who underwent surgical resection at the Second Affiliated Hospital, Zhejiang University School of Medicine. Autologous peripheral blood was collected before surgery. Normal autologous tissue was obtained from a macroscopically normal part of the excised pulmonary lobe, at least 5 cm away from the tumor. None of the patients had received radiotherapy or chemotherapy before operation.

### Tumor cell lines

A549 (human lung carcinoma) Cells were grown in Roswell Park Memorial Institute (RPMI) 1640 growth medium supplemented with 10% fetal bovine serum (FBS), 1% penicillin and streptomycin at 37 °C with 5% CO_2_ and maintained at a confluence of 70–80%.

### Cell preparations

Freshly excised tissues were cut into small pieces and then digested in RPMI 1640 medium containing 2% FBS, type I collagenase (1 mg/ml), type IV collagenase (1 mg/ml) and hyaluronidase (10 ng/ml) for 1 h at 37 °C. PB lymphocytes collected after lysing on lysing solution (BD Pharm Lyse™).

### Antibodies and flow cytometry

The antibody CD3 (UCHT1), CD8a (RPA-T8), CD38 (HB-7), CD101 (BB27), PD-1 (EH12.2H7), CD103 (Ber-ACT8), IFN-γ (B27), TNF-α (MAb11), Granzyme B (QA16A02), perforin (dG9), CD69 (FN50), HLA-DR (L243) were purchased from Biolegend. We use mechanic dispersion and enzymatic digestion to prepare the single cell of normal and tumor tissues for extracellular staining of immune markers. For blocking nonspecific binding and stained with different combinations of fluorochrome-coupled antibodies, we preincubated fresh tissue cells (1 × 10^6^/ml) in a mixture of PBS, 2% fetal calf serum and 0.1% (w/v) sodium azide with FcgIII/IIR-specific antibody. And then, we followed the manufacturer’s protocol after 12 h incubation in the presence of Leukocyte Activation Cocktail (BD Pharmingen) to perform the intracellular staining. Fluorescence data were collected on a FACSCanto II system (BD Biosciences) and analyzed using FlowJo software (Tree Star).

### In vitro culture and cell isolation

To investigate the cytokine secretion of tumor-infiltrating CD8+ T cells, single cells of normal and tumor tissues were cultured in the presence of anti-CD3 (1 ug/ml, Biolegend) and anti-CD28 (1 ug/ml, Biolegend) or PMA and ionomycin or anti-PD-1 (10 ug/ml, Biolegend). After a while, single cells of normal and tumor tissues were collected for the perforin, Granzyme B, TNF-α and IFN-γ assay.

To visualize the tumor-killing power of CD3+ CD8+ CD38+ T cells, CD3+ CD8+ CD38+ T cells and CD3+ CD8+ CD38− T cells sorted by Aria II cell sorter (BD Biosciences) and 7-AAD was used to delete dead cells. The purity of all sorted cells was greater than 90%. The sorted cells were resuspended in a 96-well plate with 2 W medium per well. After standing for 24 h, the A549 cell line was added for co-cultivation according to E (effector cell):T (tumor cell line) = 2:1. After 6 h, use 7-AAD to detect the number of dead cells in A549.

### Immunohistochemical and immunofluorescent staining

Paraffin-embedded and formalin-fixed samples were cut into 5-μM sections, which were then processed for IF staining or IHC staining. First incubation with the CD103 (ABCAM) antibody, followed by HRP conjugated Goat Anti-Mouse IgG (Servicebio). Then, incubation with antibodies against human CD8, CD38 (ABCAM), followed by Cy5 conjugated Goat Anti-mouse IgG, FITC conjugated Goat Anti-Rabbit IgG (Servicebio). Images were acquired with a confocal microscopy (Zeiss LSM 710, Carl Zeiss, Dublin, CA).

### Statistical analysis

The results were expressed as means ± SEM. Statistical analysis was using GraphPad Prism software version 6.1 to perform. The statistical significance of differences between groups was determined by the Student’s t test. All data were analyzed using two-tailed tests unless otherwise specified, and we use the *p* value < 0.05 as statistically significant.

## Results

### Result1: CD38 expression correlate with clinical prognosis and CD8+ T cell infiltration in NSCLC

**Fig. 1:**
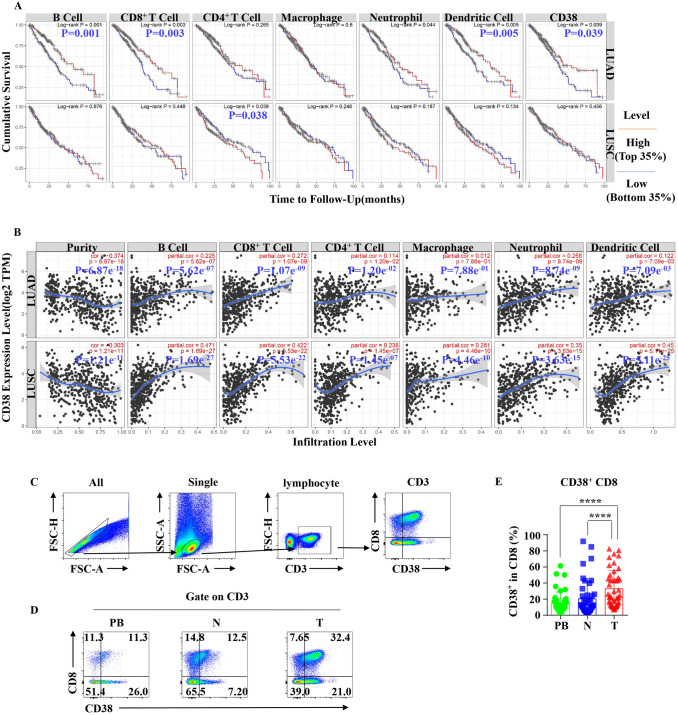
Correlation between CD38 expression and survival, CD+ CD3+ T cells infiltration in human NSCLC. **a** CD38 expression is significantly negatively related to tumor purity and has significant positive correlations with infiltrating levels of B cells, CD+ T cells, CD4+ T cells, macrophages, neutrophils and dendritic cells in LUAD, reproduced from TCGA database were determined by TIMER (Upper). And CD38 expression is significantly negatively related to tumor purity and has significant positive correlations with infiltrating levels of B cells, CD+ T cells, CD4+ T cells, macrophages, neutrophils and dendritic cells in LUSC, reproduced from TCGA database were determined by TIMER (Lower). **b** Prognostic roles of CD38 and immune-related factors in LUAD, reproduced from TCGA database were determined by TIMER (Upper). And prognostic roles of CD38 and immune-related factors in LUSC, reproduced from TCGA database were determined by TIMER (Lower). **c** Representative gating strategy for the flow cytometric analysis of CD3+ CD+ T cells and CD38− CD+ T cells in NSCLC. **d** Representative flow cytometric analysis of the expression of CD8 and CD38 by CD3+ T cells in NSCLC. **e** Bar diagram summarizes the percentage of CD3+ CD+ T cells in CD8. Data are shown as the mean ± SEM; PB = 41, *N* = 45, *T* = 45; ****p < 0.0001

Through analyzing the data of TIMER database, we found that B cells, CD8+ T cells, dendritic cells and CD38 high expression (Top 35%) were positive correlated with the survival of LUAD, and CD4+ T cells were positive correlated with the survival of LUSC (Fig. 1a). Moreover, CD38 expression was positive correlated with infiltrating levels of B cells, CD8+ T cells, CD4+ T cells, macrophages, neutrophils and dendritic cells in LUAD and LUSC, but negatively related to tumor purity (Fig. 1b). For further validation, we collected samples from 45 patients with NSCLC and analyzed the expression of CD38 in CD8+ T cells by flow cytometry (Fig. 1c, d). The information of the patients was shown in supplementary Table 1. In accordance with expectation, we found the expression level of CD38 in CD8+ T cells and the proportion of CD38+ CD8+ T cells in T cells were significantly increased in tumor (T) compared with paired normal lung tissues (N) and peripheral blood (PB) (Fig. 1e). These findings suggest that NSCLC patients with increased expression level of CD38 have a better clinical prognosis, and the CD38+ CD8+ T cells enriched in the tumor, are likely to be one of important candidates involved in anti-tumor-immune response.

### Result2: Tumor-infiltrating CD38+ CD8+ T cells are advantage in cytotoxicity, cytokine secretion and tumor-killing in vitro without additional activation

**Fig. 2:**
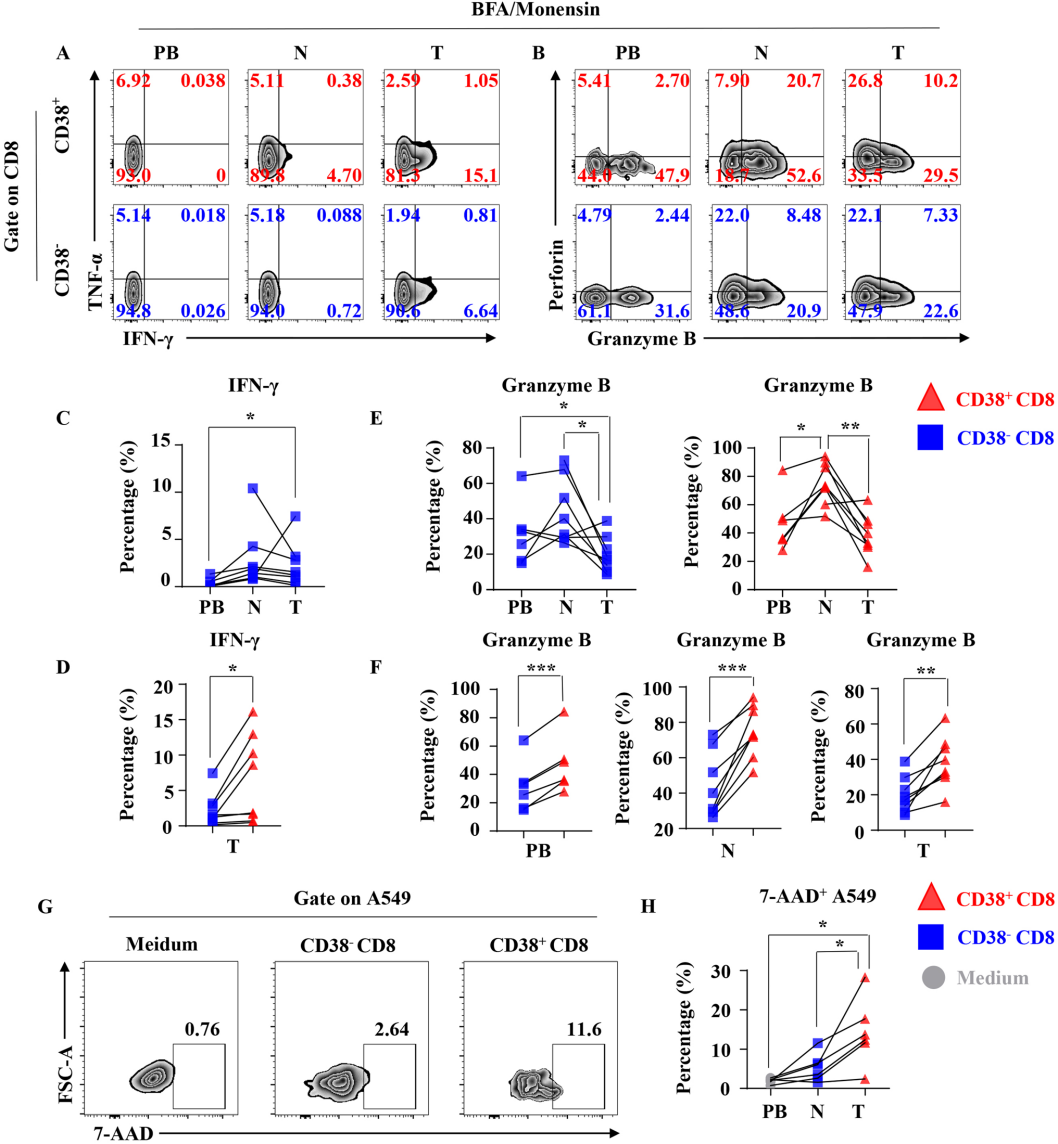
Cytotoxic molecule, cytokine secretion and tumor-killing effect of CD+ T cell subsets. **a** Representative flow cytometric analysis of the intracellular IFN-γ and TNF-α produced by CD3+ CD+ T cells (Upper) and CD38− CD+ T cells (Lower) with BFA and Monensin in NSCLC. **b** Representative flow cytometric analysis of the intracellular Granzyme B and perforin produced by CD3+ CD+ T cells (Upper) and CD38− CD+ T cells (Lower) with BFA and Monensin in NSCLC. **c** The line chart summarizes the levels of intracellular IFN-γ produced by CD38− CD+ T cells with BFA and Monensin between different organizations. Data are shown as the mean ± SEM; PB = 6, *N* = 8, *T* = 8; *p < 0.05. **d** The line chart summarizes the levels of intracellular IFN-γ produced by CD3+ CD+ T cells and CD38− CD+ T cells with BFA, Monensin in T. Data are shown as the mean ± SEM; *T* = 8; *p < 0.05. **e** The line chart summarizes the levels of intracellular Granzyme B produced by CD3+ CD+ T cells (Right) and CD38− CD+ T cells (Left) with BFA and Monensin between different organizations. Data are shown as the mean ± SEM; PB = 6, *N* = 8, *T* = 8; *p < 0.05; **p < 0.01. **f** The line chart summarizes the levels of intracellular Granzyme B produced by CD3+ CD+ T cells and CD38− CD+ T cells with BFA and Monensin in PB (Left), *N* (Middle) and *T* (Right). Data are shown as the mean ± SEM; PB = 6, *N* = 8, *T* = 8; **p < 0.01; ***p < 0.001. **g** A549 cells were co-cultured with CD3+ CD+ T cells and CD38− CD+ T cells, at 1:2 ratios, or nothing as positive control. After 6 h, culture supernatants were collected and the percentage of A549 cells dying was analyzed by flow cytometry. Representative flow cytometric shows the expression of 7-AAD by A549 co-cultivated with nothing, CD38− CD+ T cells or CD3+ CD+ T cells. **h** The line chart summarizes the expression of 7-AAD by A549. Data are shown as the mean ± SEM; *T* = 6; *p < 0.05

To further investigate the cytotoxicity and cytokine secretion of CD38+ CD8+ T cells and CD38− CD8+ T cells in natural condition, we cultured single cell suspensions from peripheral blood (*n* = 6), adjacent normal lung tissues (*n* = 8) and tumor (*n* = 8) of NSCLC patients without TCR agonist in vitro and detected the secretion of cytotoxic molecules and cytokines through flow cytometry (Fig. 2a, b). There is no significant difference in the ability to secrete TNF-α between CD38+ CD8+ T cells and CD38− CD8+ T cells in tumor (T), paired normal lung tissues (N) and peripheral blood (PB) (Supplement Figure 1A, B). Tumor-infiltrating CD38− CD8+ T cells had a stronger IFN-γ secretion capacity than that in peripheral blood, but not CD38+ CD8+ T cells (Fig. 2c and Supplement Figure 1C). It is interesting that CD38+ CD8+ T cells in tumor had a stronger secretion of IFN-γ than CD38− CD8+ T cells, but not in normal tissues and peripheral blood (Fig. 2d and Supplement Figure 1D). There is no significant difference in perforin secretion between CD38+ CD8+ T cells and CD38− CD8+ T cells in all tissues (Supplement Figure 1E, F). Comparing with peripheral blood and tumors, the expression level of Granzyme B was higher in both CD38− CD8+ T cells and CD38+ CD8+ T cells (Fig. 2e). However, no matter which tissue it is derived from, the secretion capacity of Granzyme B was significant higher in CD38+ CD8+ T cells than CD38− CD8+ T cells (Fig. 2f). Furthermore, using a co-cultured experiment in vitro (Supplement Figure 2A, B, C, D), we found that tumor-infiltrating CD38+ CD8+ T cells had a stronger tumor-killing effect than CD38− CD8+ T cells (Fig. 2g, h). Taken together, despite the universal decreasing tendency of cytotoxicity and cytokine secretion of CD38+ CD8+ T cells and CD38− CD8+ T cells in tumor compared with normal tissue, our results found that tumor-infiltrating CD38+ CD8+ T cells are advantages in cytotoxicity, cytokine secretion and tumor-killing ability than tumor-infiltrating CD38− CD8+ T cells in the natural condition which may result from pre-activation.

### Result3: Tumor-infiltrating CD38+ CD8+ T cells are advantage in the secretion of IFN-γ and Granzyme B in activated condition

**Fig. 3:**
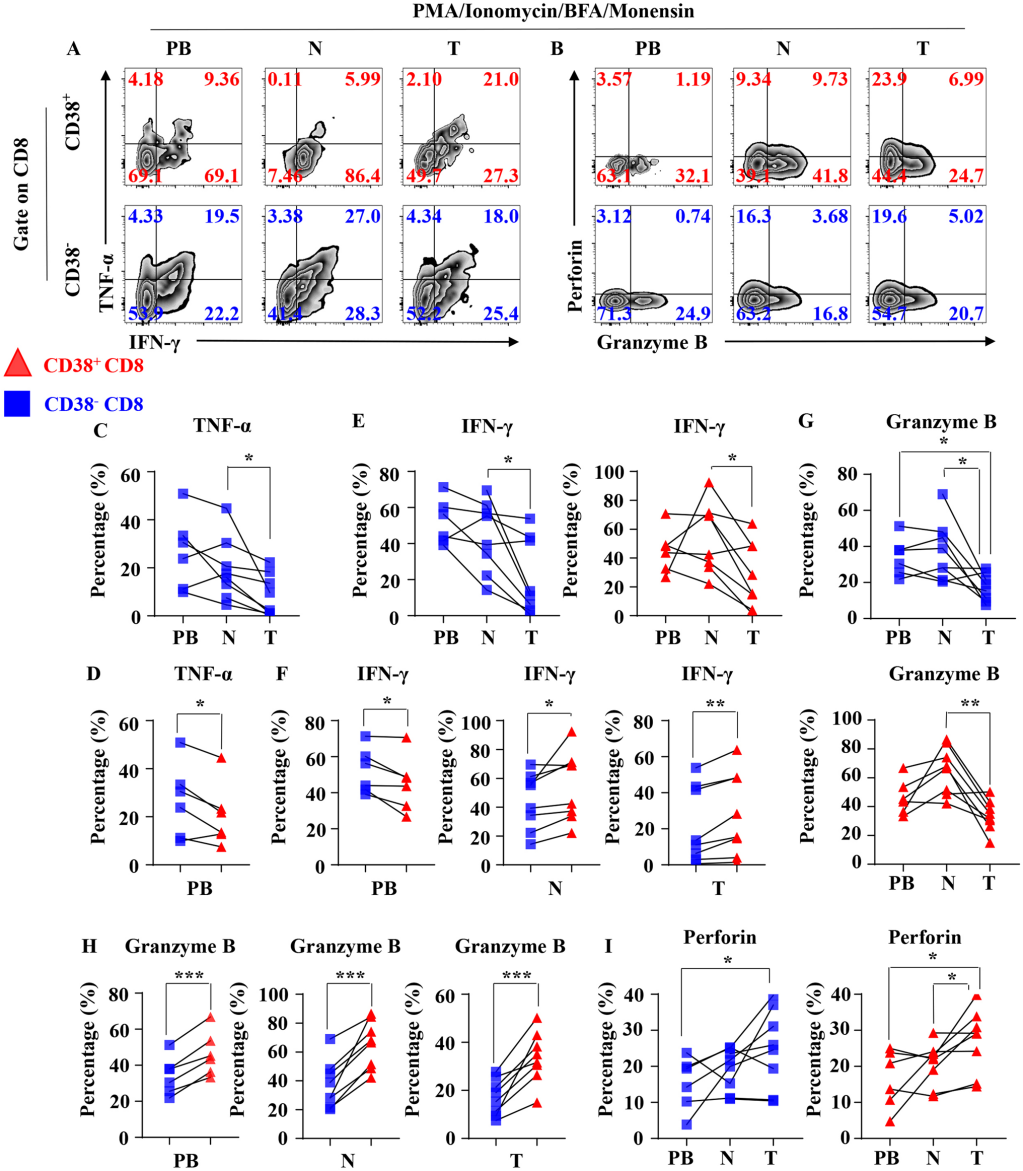
Cytotoxic molecule and cytokine secretion of reactivated CD+ T cell subsets. **a** Representative flow cytometric analysis of the intracellular IFN-γ and TNF-α produced by CD3+ CD+ T cells (Upper) and CD38− CD+ T cells (Lower) with PMA, Ionomycin, BFA and Monensin in NSCLC. **b** Representative flow cytometric analysis of the intracellular Granzyme B and perforin produced by CD+ CD3+ T cells (Upper) and CD+ CD38− T cells (Lower) with PMA, Ionomycin, BFA and Monensin in NSCLC. **c** The line chart summarizes the levels of intracellular TNF-α produced by CD38− CD+ T cells with PMA, Ionomycin, BFA, Monensin between different organizations. Data are shown as the mean ± SEM; PB = 6, *N* = 8, *T* = 8; *p < 0.05. **d** The line chart summarizes the levels of intracellular TNF-α produced by CD3+ CD+ T cells and CD38− CD+ T cells with PMA, Ionomycin, BFA, Monensin in PB. Data are shown as the mean ± SEM; PB = 6; *p < 0.05. **e** The line chart summarizes the levels of intracellular IFN-γ produced by CD3+ CD+ T cells (Right) and CD38− CD+ T cells (Left) with PMA, Ionomycin, BFA and Monensin between different organizations. Data are shown as the mean ± SEM; PB = 6, *N* = 8, *T* = 8; *p < 0.05. **f** The line chart summarizes the levels of intracellular IFN-γ produced by CD3+ CD+ T cells and CD38− CD+ T cells with BFA and Monensin in PB (Left), *N* (Middle) and *T* (Right). Data are shown as the mean ± SEM; PB = 6, N = 8, T = 8; *p < 0.05; **p < 0.01. **g** The line chart summarizes the levels of intracellular Granzyme B produced by CD3+ CD+ T cells (Upper) and CD38− CD+ T cells (Lower) with PMA, Ionomycin, BFA and Monensin between different organizations. Data are shown as the mean ± SEM; PB = 6, *N* = 8, *T* = 8; *p < 0.05; **p < 0.01. **h** The line chart summarizes the levels of intracellular Granzyme B produced by CD3+ CD+ T cells and CD38− CD+ T cells with PMA, Ionomycin, BFA and Monensin in PB (Peripheral Blood, Left), *N* (Normal tissue, Middle) and *T* (Tumor, Right). Data are shown as the mean ± SEM; PB = 6, *N* = 8, *T* = 8; ***p < 0.001. **i** The line chart summarizes the levels of intracellular perforin produced by CD3+ CD+ T cells (Right) and CD38− CD+ T cells (Left) with PMA, Ionomycin, BFA and Monensin between different organizations. Data are shown as the mean ± SEM; PB = 6, *N* = 8, *T* = 8; *p < 0.05

Next, we investigated the cytotoxicity, cytokine secretion of CD38+ CD8+ T cells and CD38− CD38+ T cells in the fully activated condition induced by PMA and Ionomycin (Fig. 3a, b). We found that the TNF-α secretion in tumor-infiltrating CD38− CD8+ T cells was significantly decreased when compare with CD38− CD8+ T cells in paired normal tissues (Fig. 3c), but not in tumor-infiltrating CD38+ CD8+ T cells (Supplement Figure 3A). In peripheral blood, the TNF-α secretion capacity of CD38− CD8+ T cells was stronger than that of CD38+ CD8+ T cells (Fig. 3d), but not in normal tissues or tumors (Supplement Figure 2B). In terms of the secretion capacity of IFN-γ, CD38+ CD8+ T cells and CD38− CD8+ T cells in tumor were decreased in IFN-γ secretion than their counterparts in normal tissues (Fig. 3e). It was also interesting that the IFN-γ secretion of CD38+ CD8+ T cells from peripheral blood was weaker than that of CD38− CD8+ T cells, while the secretion of IFN-γ from normal tissues and tumors was stronger than that of CD38− CD8+ T cells (Fig. 3f). Then, we found tumor-infiltrating CD38+ CD8+ T cells and CD38− CD8+ T cells both were decreased in the secretion of Granzyme B compared with normal tissue and peripheral blood (Fig. 3g). Consistent with previous results, the Granzyme B secretion capacity of CD38+ CD8+ T cells was stronger than that of CD38− CD8+ T cells in all types of tissue (Fig. 3h). CD38+ CD8+ T cells and CD38− CD8+ T cells showed advantages in secrete perforin compared with their counterparts in both normal tissue and peripheral blood (Fig. 3i, Supplement Figure 2C). However, there was no difference between the perforin secretion between tumor-infiltrating CD38+ CD8+ T cells and tumor-infiltrating CD38− CD8+ T cells (Supplement Figure 2C). These results verified the advantages of tumor-infiltrating CD38+ CD8+ T cells in IFN-γ and Granzyme B secretion than tumor-infiltrating CD38− CD8+ T cells in fully activated condition.

### Result4: Tumor-infiltrating CD38+ CD8+ T cells show a dual phenotype when compared with CD38− CD8+ T cells

**Fig. 4:**
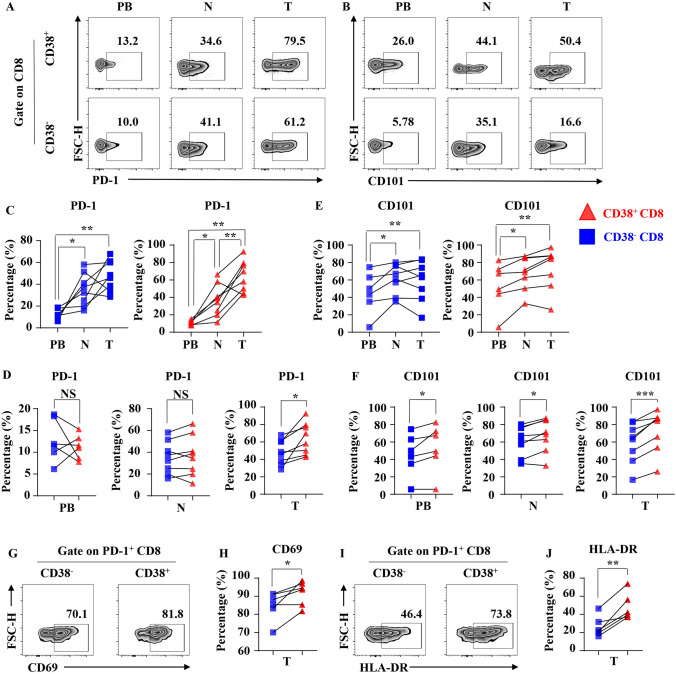
Markers of T cell exhaustion and activation express in tumor-infiltrating CD3+ CD+ T cells. **a** Representative flow cytometric analysis of the expression of PD-1 by CD3+ CD+ T cells and CD38− CD+ T cells in NSCLC. **b** Representative flow cytometric analysis of the expression of CD101 by CD3+ CD+ T cells and CD38− CD+ T cells in NSCLC. **c** The line chart summarizes the levels of PD-1 by CD3+ CD+ T cells (Right) and CD38− CD+ T cells (Left) in NSCLC between different organizations. Data are shown as the mean ± SEM; PB = 6, *N* = 8, *T* = 8; *p < 0.05; **p < 0.01. **d** The line chart summarizes the levels of PD-1 by CD3+ CD+ T cells and CD38− CD+ T cells in NSCLC in PB (Left), *N* (Middle) and *T* (Right). Data are shown as the mean ± SEM; PB = 6, N = 8, T = 8; NS *p* ≥ 0.05; *p < 0.05. **e** The line chart summarizes the levels of CD101 by CD3+ CD+ T cells (Right) and CD38− CD+ T cells (Left) in NSCLC between different organizations. Data are shown as the mean ± SEM; PB = 6, N = 8, T = 8; *p < 0.05; **p < 0.01. **f** The line chart summarizes the levels of CD101 by CD3+ CD+ T cells and CD38− CD+ T cells in NSCLC in PB (Left), N (Middle) and T (Right). Data are shown as the mean ± SEM; PB = 6, N = 8, T = 8; *p < 0.05; ***p < 0.001. **g** Representative flow cytometric shows the expression of CD69 by PD-1+ CD3+ CD+ T cells and PD-1+ CD38− CD+ T cells in NSCLC. **h** The line chart summarizes the levels of CD69 by CD3+ CD+ T cells and CD38− CD+ T cells in NSCLC. Data are shown as the mean ± SEM; T = 6; *p < 0.05. **i** Representative flow cytometric shows the expression of HLA-DR by PD-1+ CD3+ CD+ T cells and PD-1+ CD38− CD+ T cells in NSCLC. **j** The line chart summarizes the levels of HLA-DR by CD3+ CD+ T cells and CD38− CD+ T cells in NSCLC. Data are shown as the mean ± SEM; *T* = 6; **p < 0.01

In our study, we also found that tumor-infiltrating CD38+ CD8+ T cells and CD38− CD8+ T cells have significantly decreased of IFN-γ and Granzyme B secretion compared to adjacent normal tissues (Figs. 2e and 3e, g). We proposed that the functional impairment of CD38+ CD8+ T cells in tumor might be related to the occurrence of exhaustion. To verify our hypothesis, we analyzed the expression of PD-1 and CD101 in tumor-infiltrating CD38+ CD8+ T cells and CD38− CD8+ T cells by flow cytometry (Fig. 4a, b). We found that the expression of PD-1 significantly increased in tumor-infiltrating CD38+ CD8+ T cells compared with adjacent normal tissues and peripheral blood (Fig. 4c). While in CD38− CD8+ T cells, the expression of PD-1 in tumor and normal tissues was higher than that in peripheral blood, but not increased in tumor-infiltrating CD38− CD8+ T cells compared with CD38− CD8+ T cells in normal tissue (Fig. 4c). Moreover, the increased expression of PD-1 in CD38+ CD8+ T cells compared with CD38− CD+ T cells was only observed in tumor, but not in peripheral blood and normal tissues (Fig. 4d). In addition, CD3+ CD+ T cells and CD38− CD+ T cells derived from tumors and normal tissues had higher expression of CD101 than those derived from peripheral blood (Fig. 4e). Moreover, in all types of tissues, the CD101 expression of CD3+ CD+ T cells is higher than that of CD38− CD+ T cells (Fig. 4f).

Next, we compared the degree of activation of tumor-infiltrating PD-1+ CD3+ CD+ T cells with tumor-infiltrating PD-1+ CD38− CD+ T cells using CD69 and HLA-DR (Fig. 4g, i), which are the markers of T cell activation. It is interesting that tumor-infiltrating PD-1+ CD3+ CD+ T cells expressed higher levels of both CD69 and HLA-DR than tumor-infiltrating PD-1+ CD38− CD+ T cells (Fig. 4h, j). These findings suggest that CD3+ CD+ T cells in tumor of NSCLC had an exhausted phenotype which may result from the advantages in pre-activation.

### Result5: the expression of CD103 in CD3+ CD+ T cells is increased in tumor and can be induced by activation in vitro

**Fig. 5:**
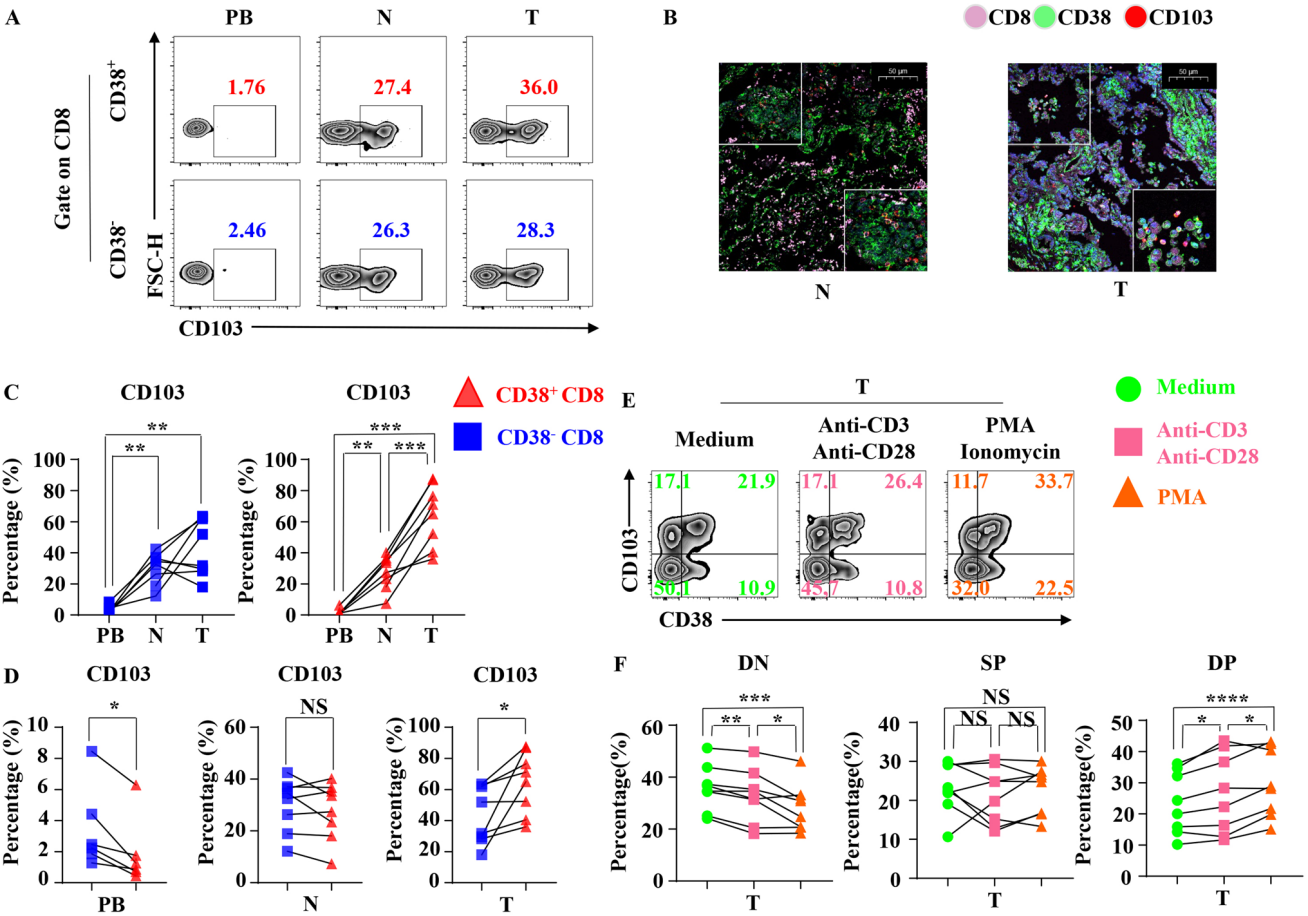
CD103 expression of tumor-infiltrating CD3+ CD+ T cells. **a** Representative flow cytometric analysis of the expression of CD103 by CD3+ CD+ T cells and CD38− CD+ T cells in NSCLC. **b** Paraffin sections from lung cancer patients were stained with antihuman CD103 (red), CD38 (green) and antihuman CD8 (pink) for immunofluorescent (IF) staining. One of six independent experiments is shown. N (healthy normal tissue adjacent to the tumor, *n* = 6) and T (tumor, *n* = 6). **c** The line chart summarizes the levels of CD103 by CD3+ CD+ T cells (Right) and CD38− CD+ T cells (Left) in NSCLC between different organizations. Data are shown as the mean ± SEM; PB = 6, *N* = 8, *T* = 8; **p < 0.01; ***p < 0.001. **d** The line chart summarizes the levels of CD103 by CD3+ CD+ T cells and CD38− CD+ T cells in NSCLC in PB (Left), *N* (Middle) and *T* (Right). Data are shown as the mean ± SEM; PB = 6, *N* = 8, *T* = 8; NS *p* ≥ 0.05; *p < 0.05. **e** Representative flow cytometric analysis of the expression of CD38 and CD103 by CD+ T cells with BFA and Monensin (Left) or with BFA, Monensin, anti-CD3 (1 ug/ml), anti-CD28 (1 ug/ml) (Middle) or with BFA, Monensin, PMA, and Ionomycin (Right) in NSCLC. **f** The line chart summarizes the percentage change of DN (CD38− CD103− CD+ T cells, Left), SP (CD3+ CD103− CD+ T cells, Middle), DP (CD3+ CD103+ CD+ T cells, Right) with BFA and Monensin or with BFA, Monensin, anti-CD3 (1 ug/ml), anti-CD28 (1 ug/ml) or with BFA, Monensin, PMA, and Ionomycin in NSCLC. Data are shown as the mean ± SEM; *T* = 8; NS *p* ≥ 0.05; *p < 0.05; **p < 0.01; ***p < 0.001; ****p < 0.0001

The tissue-resident CD+ T cell, mainly identified by the CD103 marker, is an important subgroup exerting anti-tumor efficacy in tumor-immune microenvironment. In order to further understand the reasons for superior functional performance of CD3+ CD+ T cells, we analyzed the expression of CD103 on CD3+ CD+ T cells by flow cytometry and immunofluorescence (Fig. 5a, b). We found the number of CD3+ CD103+ CD+ T cells highly increased in tumors and were significantly higher than normal tissues and peripheral blood, while in CD38− CD+ T cells, the expression of CD103 in normal tissues and tumor were higher than that from peripheral blood, but there is no difference between the two (Fig. 5c, d). Moreover, immunofluorescence assay also showed the same results (Fig. 5b). Then, we activated tumor-infiltrating CD+ T cells through anti-CD3, anti-CD28, PMA and Ionomycin in vitro for 12 h (Fig. 5e). We found that CD3+ CD103+ CD+ T cells increased significantly after activation, and CD38− CD103− CD+ T cells significantly decreased in contrast. Meanwhile, the proportion of CD3+ CD103− CD+ T cells did not change (Fig. 5f). These results indicated that the increased CD103 expression in CD3+ CD+ T cells can be induced by activation and sustains their accumulation.

### Result6: CD103+ CD3+ CD+ T cells secrete higher IFN-γ, TNF-α and perforin in tumor and can be reinvigorated by anti-PD-1 in vitro

**Fig. 6:**
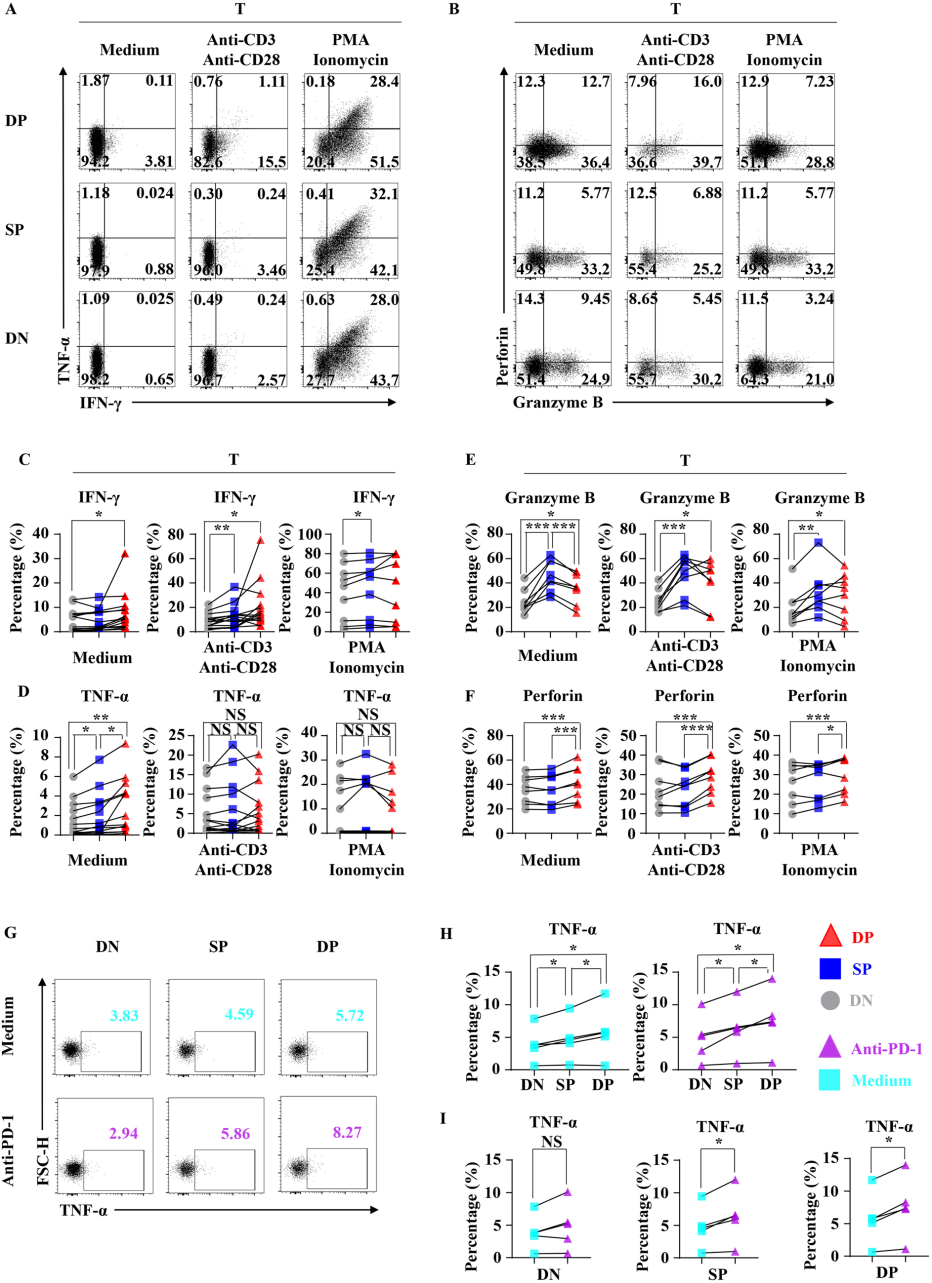
Anti-tumor cytokine profiling of tumor-infiltrating CD+ T cell subsets according to CD38 and CD103 expression. **a** Representative flow cytometric analysis of the intracellular IFN-γ and TNF-α produced by DN (CD38− CD103− CD+ T cells, Lower), SP (CD3+ CD103− CD+ T cells, Middle), DP (CD3+ CD103+ CD+ T cells, Upper) with BFA and Monensin (Left) or with BFA, Monensin, anti-CD3 (1 ug/ml), anti-CD28 (1 ug/ml) (Middle) or with BFA, Monensin, PMA, and Ionomycin (Right) in NSCLC. **b** Representative flow cytometric analysis of the intracellular Granzyme B and perforin produced by DN (Lower), SP (Middle), DP (Upper) with BFA and Monensin (Left) or with BFA, Monensin, anti-CD3 (1 ug/ml), anti-CD28 (1 ug/ml) (Middle) or with BFA, Monensin, PMA, and Ionomycin (Right) in NSCLC. **c** Bar diagram summarizes the levels of intracellular IFN-γ produced by DN (Left), SP (Middle), DP (Right). Data are shown as the mean ± SEM; *T* = 14 (Medium, anti-CD3 and anti-CD38), or 9(PMA and Ionomycin); *p < 0.05; **p < 0.01. **d** Bar diagram summarizes the levels of intracellular TNF-α produced by DN (Left), SP (Middle), DP (Right). Data are shown as the mean ± SEM; *T* = 14 (Medium, anti-CD3 and anti-CD38), or 9(PMA and Ionomycin); NS *p* ≥ 0.05; *p < 0.05; **p < 0.01. **e** The line chart summarizes the levels of intracellular Granzyme B produced by DN (Left), SP (Middle), DP (Right). Data are shown as the mean ± SEM; *T* = 8; *p < 0.05; **p < 0.01; ***p < 0.001. **f** The line chart summarizes the levels of intracellular perforin produced by DN (Left), SP (Middle), DP (Right). Data are shown as the mean ± SEM; T = 8; *p < 0.05; ***p < 0.001; ****p < 0.0001. **g** Representative flow cytometric analysis of the intracellular TNF-α produced by DN (Left), SP (Middle), DP (Right) with BFA and Monensin or BFA, Monensin, anti-PD-1 (10 ug/ml) in NSCLC. **h** and **i** The line chart summarizes the levels of intracellular TNF-α produced by DN, SP, DP with BFA and Monensin or BFA, Monensin, anti-PD-1 (10 ug/ml) in NSCLC. Data are shown as the mean ± SEM; *T* = 5; NS *p* ≥ 0.05; *p < 0.05

To further investigate the impact of CD103 expression on tumor-infiltrating CD3+ CD+ T cells, we stimulated tumor-infiltrating CD+ T cells through anti-CD3, anti-CD28, PMA and Ionomycin or anti-PD-1 in vitro for 12 h (Fig. 6a, b). We found that CD3+ CD103+ CD+ T cells secrete more IFN-γ compared with CD38− CD103− CD+ T cells without stimulation or with anti-CD3, anti-CD28 (Fig. 6c). And, CD3+ CD103+ CD+ T cells had a stronger ability to secrete TNF-α than both CD3+ CD103− CD+ T cells and CD38− CD103− CD+ T cells (Fig. 6d). About the Granzyme B expression, CD3+ CD103+ CD+ T cells and CD3+ CD103− CD+ T cells were higher than CD38-CD103− CD+ T cells (Fig. 6e). As for perforin, the secretion capacity in CD3+ CD103+ CD+ T cells was higher than CD3+ CD103− CD+ T cells and CD38− CD103− CD+ T cells (Fig. 6f). Intriguingly, CD3+ CD103+ CD+ T cells and CD3+ CD103− CD+ T cells can both be reinvigorated by anti-PD-1, and CD3+ CD103+ CD+ T cell showed stronger response (Fig. 6g–i). These results suggested that increased CD103 expression may sustain the cytotoxicity and cytokine secretion of tumor-infiltrating CD3+ CD+ T cells as well as response to anti-PD-1.

## Discussion

While curative tumor resection remains the most effective therapy for NSCLC, accumulating evidence has suggested various patients benefits from pre- or postoperative immunotherapy tremendously [18]. Unfortunately, PD-1/PD-L1 antibody, the most popular strategy for immunotherapy treatment, fails to show universal advantage in NSCLC patients, which is presumably attributed to volatile tumor-immune microenvironment [19]. Therefore, it is urgent to uncover the underlying mechanism and specific immune cell types exerting anti-tumor effect, in the hope of screening out patients suitable for immunotherapy more effectively, and consequently improving prognosis in NSCLC patients.

Among multiple immune cells, CD+ T cells have attracted growing concentration for their obvious activity during tumor-killing process [20]. Meanwhile, CD+ T cells can be further classified into subgroups based on cell surface markers, and the expression of cell surface receptor CD38 has been widely considered a vital symbol of CD+ T cell activation. CD38 is highest expressed on the surface of NK cells, followed by subpopulations of B and T cells [11, 21]. It was reported to be involved in triggering activation and proliferation signals that are lineage-unrestricted [22]. Although CD38 displays receptor and enzymatic activities that contribute to the establishment of an effective immune response, some works raise the possibility that CD38 might also enhance the immunosuppressive potential of regulatory leukocytes [11]. Therefore, what immunological role it plays in the NSCLC tumor microenvironment has aroused our interest.

In our research, we first evaluated the association of CD38 expression with NSCLC prognosis and immune infiltration levels separately through the TIMER public database. Our online analysis proved increased CD38 expression exhibited a better clinical prognosis and has a significant correlation with CD+ T cells in NSCLC. Evidence above inspired us to focus on the role of CD3+ CD+ T cells in NSCLC tumor microenvironment. Through further study on clinical specimens from NSCLC patients, we found that CD3+ CD+ T cells highly accumulated in tumor. This indicated the subset of T cells might play a crucial role in anti-tumor immunity of NSCLC patients. Our hypothesis was further confirmed in subsequent experiments with primary cells in vitro, which showed CD3+ CD+ T cells indeed have a stronger ability to secrete cytotoxic factors than CD38− CD+ T cells in paired tumors or normal lung tissues. Furthermore, through co-cultivation with A549, we directly proved that tumor-infiltrating CD3+ CD+ T cells have a more strong tumor-killing capacity than tumor-infiltrating CD38− CD+ T cells in vitro. It is interesting that we found opposite result in the peripheral blood, where CD3+ CD+ T cells isolated from peripheral blood were less effective in cytotoxic factors secretion, such as TNF-α and INF-γ, than CD38− CD+ T cells isolated from peripheral blood. This might be because peripheral blood T cells receive little or no tumor antigen stimulation compared with tumor tissues, so there exist such differences in the function of this group of cells [23].

What’s more, another interesting result attracted our attention. The tumor-infiltrating CD3+ CD+ T cells and CD38− CD+ T cells have significantly decreased secretion of IFN-γ and Granzyme B compared to paired adjacent normal tissues. This suggests that these cells in tumor are impaired in the anti-tumor function [24]. Considering the immune cells in tumor were exposed to constant tumor antigens stimulation than that in normal tissues [23], we assume that this result may be related to the occurrence of T cell exhaustion, a state of T cell dysfunction that arises during many chronic infections and cancer [25]. Exhausted T cells always show poor effector function, sustained expression of inhibitory receptors and a transcriptional state distinct from that of functional effector or memory T cells [25]. Although the details of T cell dysfunction remain unclear, the upregulation of PD-1 on T cells has emerged as a major marker of T cell dysfunction [24]. Therefore, we examined the expression levels of PD-1 and CD101, a demonstrated marker for exerting negative-costimulatory effects [26], on these two subsets of CD+ T cells. In line with our hypothesis, we found that tumor-infiltrating CD3+ CD+ T cells expressed higher level of PD-1 and CD101 than that in normal tissues. However, despite the significantly increased expression of PD-1 and CD101, tumor-infiltrating CD3+ CD+ T cells still retained a higher ability to secrete cytotoxic-related cytokine and killing tumors. We found that tumor-infiltrating PD-1+ CD3+ CD+ T cells expressed higher CD69 and HLA-DR than tumor-infiltrating PD-1− CD3+ CD+ T cells isolated from the same samples, which suggest PD-1+ subset in tumor-infiltrating CD3+ CD+ T cells are more activated.

Another reason why CD3+ CD+ T cells have stronger killing ability may be that CD3+ CD+ T cells have stronger resident characteristics in tumor tissues. Our study also suggests that tumor-infiltrating CD3+ CD+ T cells express a high level of CD103, a demonstrated marker of TRM cells, compared with CD38− CD+ T cells [27, 28]. Increased CD103 expression facilitates T cells to reside in epithelial tissue via the interaction between CD103 and E-cadherin [29]. As mentioned in the previous studies, cells with a TRM phenotype display high levels of exhaustion markers. Moreover, these cells have higher proliferative capacities and expressed molecules linked to cytotoxicity, indicating the presence of a highly activated T cell subset in CD+ tumor-infiltrating lymphocytes (TILs) [30, 31]. Here, we found that CD103+ CD3+ CD+ T cells increased significantly after activation, conversely CD103− CD38− CD+ T cells significantly decreased. These indicate that CD103+ CD3+ CD+ T cells are the main activated cells after stimulation and the tissue-resident properties of this subgroup cells are enhanced. Meanwhile, these results also suggest there may be a process of mutual transformation between the two groups, which may provide some clues for the source of TRM precursor cells. Besides, we found CD103+ CD3+ CD+ T cells secrete more IFN-γ, TNF-α and perforin than the other two subgroups in tumor. The strong functional retention of CD103+ CD3+ CD+ T cells may be related to its tissue-resident characteristics.

Another important finding in our experiment is that CD103+ CD3+ CD+ T cells secreted higher level of TNF-α to mediate an anti-tumor-immune response after stimulated by PD-1 blockade in vitro. These results suggest the impaired CD3+ CD+ T cells are able to be rescued by PD-1 blockade and also indicate CD3+ CD+ T cells may be an important subset of cells that play anti-tumor roles in anti-PD-1 immunotherapy. However, a recent study showed that CD3+ CD+ T cells without optimally priming were responsible for anti-PD-1 therapy resistance in a mouse model of metastatic melanoma and lung cancer [32]. These divergences may result from the biological differences between preexisting tumor-infiltrating CD3+ CD+ T cells in human NSCLC and CD3+ CD+ T cells induced by vaccine and anti-PD-1 therapy in mouse model of metastatic melanoma and lung cancer [32].

In conclusion, our results showed that CD3+ CD+ T cells were highly enriched in tumors. This subset of tumor-infiltrating CD+ T cells with tissue-resident characteristics and stronger anti-tumor activity are a subset of CD+ TILs with pre-activated and exhausted phenotype in NSCLC. Moreover, we also found the impaired function of tumor-infiltrating CD3+ CD+ T cells can be more effectively reinvigorated by PD-1 blockade. Therefore, tumor-infiltrating CD3+ CD+ T cell is an important anti-tumor subgroup in NSCLC and a promising candidate for future basic research. Further investigation and characterization of tumor-infiltrating CD3+ CD+ T cells would help to develop new strategies to improve the effectiveness of immunotherapy by targeting preexisting tumor-reactive CD+ T cells in in NSCLC.

### Supplementary Information

Below is the link to the electronic supplementary material.

Supplementary file1 (DOCX 12 kb)

Supplementary file2 (PDF 1023 kb)

Supplementary file3 (PDF 428 kb)

Supplementary file4 (PDF 1043 kb)

Supplementary file5 (PDF 113 kb)
