# Supplementary material for: CD38 identifies pre-activated CD8+ T cells which can be reinvigorated by anti-PD-1 blockade in human lung cancer
Source: Cancer Immunol Immunother. 2021 May 2;70(12):3603–16. doi: 10.1007/s00262-021-02949-w (PMC8571140; doi:10.1007/s00262-021-02949-w)
Supplement: Supplementary file 1 — Supplementary file1 (DOCX 12 kb) [file 262_2021_2949_MOESM1_ESM.docx]

**Supplementary Figure 1 |** **The secretion of IFN-γ, TNF-α, Granzyme B and Perforin in CD8+ T cells with BFA and Monensin.**

A. The line chart summarizes the levels of intracellular TNF-α produced by CD38+ CD8+ T cells and CD38- CD8+ T cells with BFA, Monensin between different organizations. Data are shown as the mean ± SEM; PB = 6, N = 8, T = 8; NS p ≥ 0.05.

B. The line chart summarizes the levels of intracellular TNF-α produced by CD38+ CD8+ T cells and CD38- CD8+ T cells with PMA, Ionomycin, BFA, Monensin in PB (Peripheral Blood, Left), N (Normal tissue, Middle) and T (Tumor, Right). Data are shown as the mean ± SEM; PB = 6, N = 8, T = 8; NS p ≥ 0.05.

C. The line chart summarizes the levels of intracellular IFN-γ produced by CD38+ CD8+ T cells with BFA and Monensin between different organizations. Data are shown as the mean ± SEM; PB = 6, N = 8, T = 8; NS p ≥ 0.05.

D. The line chart summarizes the levels of intracellular IFN-γ produced by CD38+ CD8+ T cells and CD38- CD8+ T cells with BFA, Monensin in PB (Left), N (Right). Data are shown as the mean ± SEM; PB = 6, N = 8; NS p ≥ 0.05.

E. The line chart summarizes the levels of intracellular Perforin produced by CD38+ CD8+ T cells and CD38- CD8+ T cells with BFA, Monensin between different organizations. Data are shown as the mean ± SEM; PB = 6, N = 8, T = 8; NS p ≥ 0.05.

F. The line chart summarizes the levels of intracellular Perforin produced by CD38+ CD8+ T cells and CD38- CD8+ T cells with BFA, Monensin in PB (Peripheral Blood, Left), N (Normal tissue, Middle) and T (Tumor, Right). Data are shown as the mean ± SEM; PB = 6, N = 8, T = 8; NS p ≥ 0.05.

**Supplementary Figure 2 | The secretion of IFN-γ, TNF-α, Granzyme B and Perforin in CD8+ T cells with PMA, Ionomycin, BFA and Monensin.**

A. The line chart summarizes the levels of intracellular TNF-α produced by CD38+ CD8+ T cells with PMA, Ionomycin, BFA and Monensin between different organizations. Data are shown as the mean ± SEM; PB = 6, N = 8, T = 8; NS p ≥ 0.05.

B. The line chart summarizes the levels of intracellular TNF-α produced by CD38+ CD8+ T cells and CD38- CD8+ T cells with PMA, Ionomycin, BFA and Monensin in PB (Peripheral Blood, Left), N (Normal tissue, Middle) and T (Tumor, Right). Data are shown as the mean ± SEM; N = 8, T = 8; NS p ≥ 0.05.

C. The line chart summarizes the levels of intracellular Perforin produced by CD38+ CD8+ T cells and CD38- CD8+ T cells with PMA, Ionomycin, BFA and Monensin in PB (Peripheral Blood, Left), N (Normal tissue, Middle) and T (Tumor, Right). Data are shown as the mean ± SEM; PB = 8, N = 8, T = 8; NS p ≥ 0.05.

**Supplementary Figure 3 | Gating strategy of co-culture experiment.**

A. Representative gating strategy for the flow cytometric sorting of CD38+ CD8+ T cells and CD38- CD8+ T cells in NSCLC.

B Representative gating strategy for quality control after sorting of CD38+ CD8+ T cells and CD38- CD8+ T cells in NSCLC.

C. Representative gating strategy for the flow cytometric analysis of sorted T cells co-culture of A549 cell lines.

D. Representative gating strategy for the flow cytometric analysis of A549 cell lines.
