# Supplementary figures and images for: CD38 identifies pre-activated CD8+ T cells which can be reinvigorated by anti-PD-1 blockade in human lung cancer
Source: Cancer Immunol Immunother. 2021 May 2;70(12):3603–16. doi: 10.1007/s00262-021-02949-w (PMC8571140; doi:10.1007/s00262-021-02949-w)

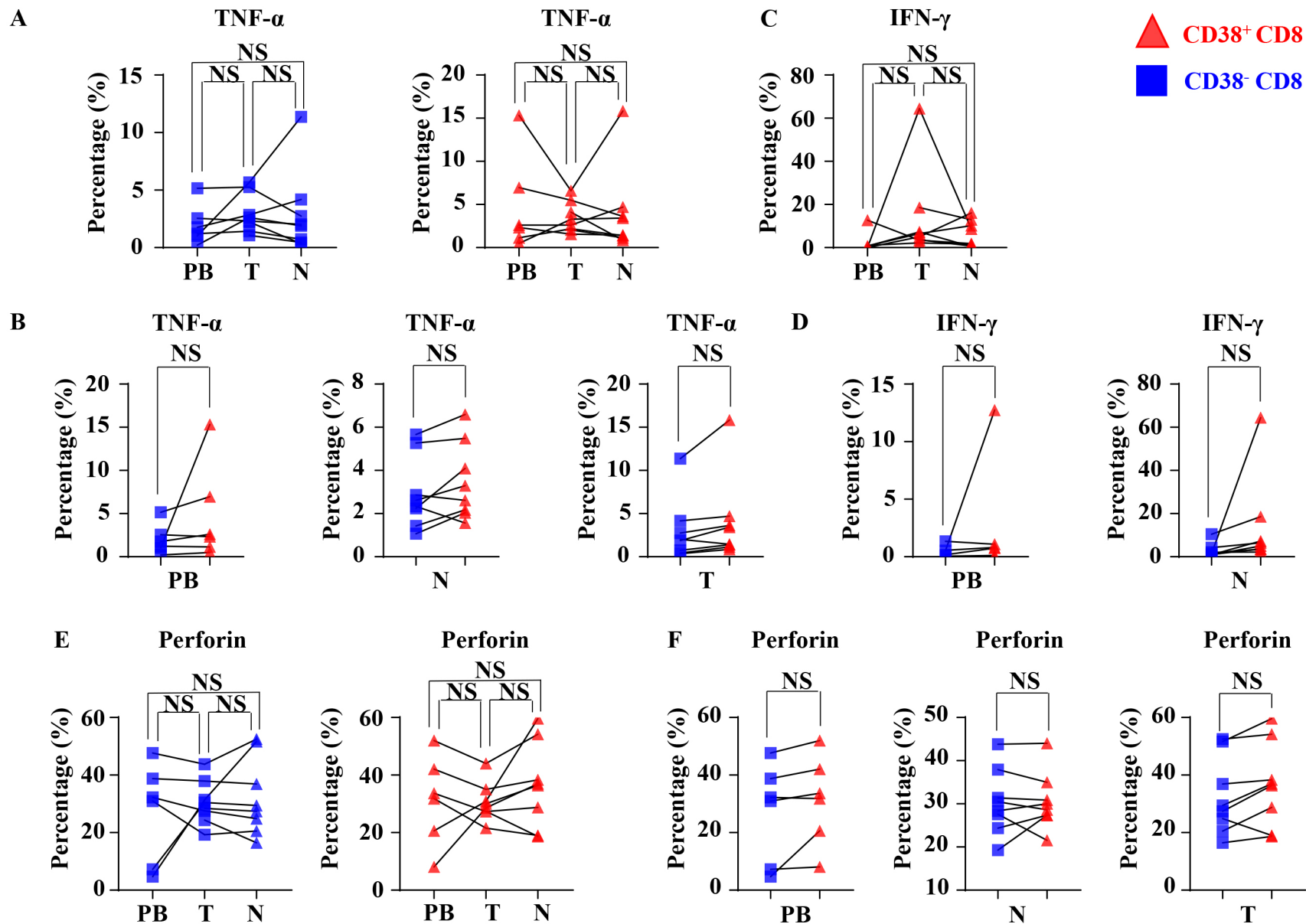

Supplement: Supplementary file 2 — Supplementary file2 (PDF 1023 kb) [file 262_2021_2949_MOESM2_ESM.pdf]

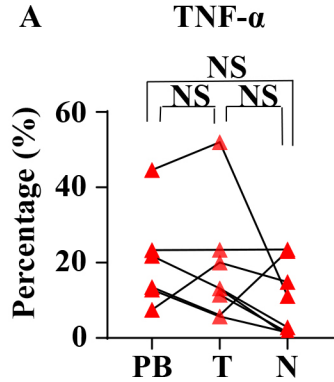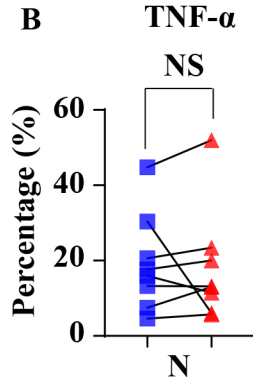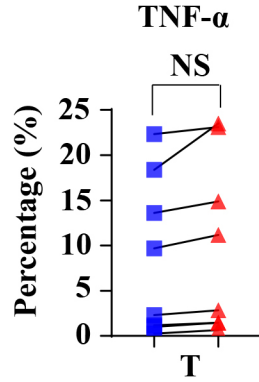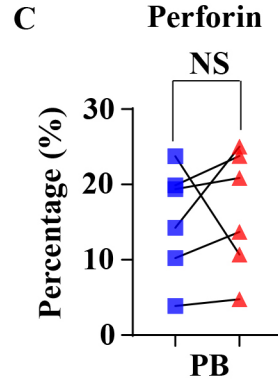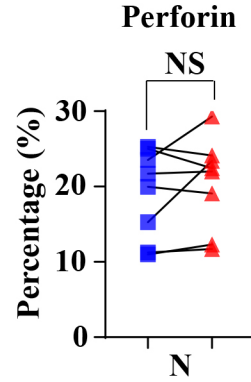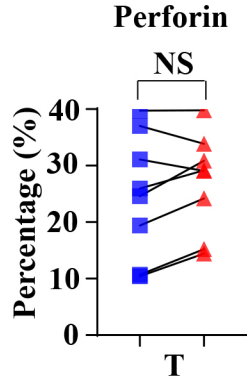

Supplement: Supplementary file 3 — Supplementary file3 (PDF 428 kb) [file 262_2021_2949_MOESM3_ESM.pdf]

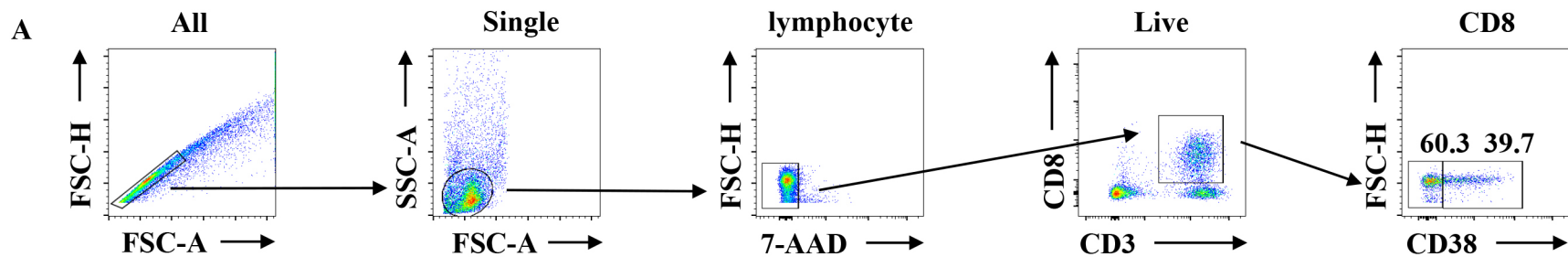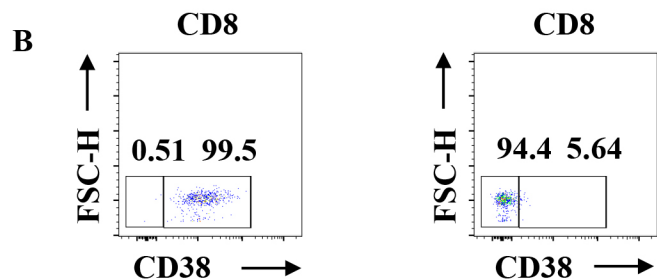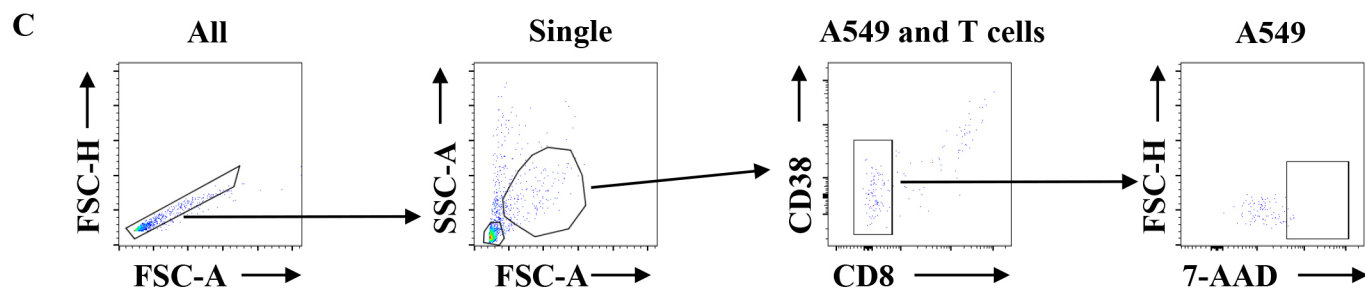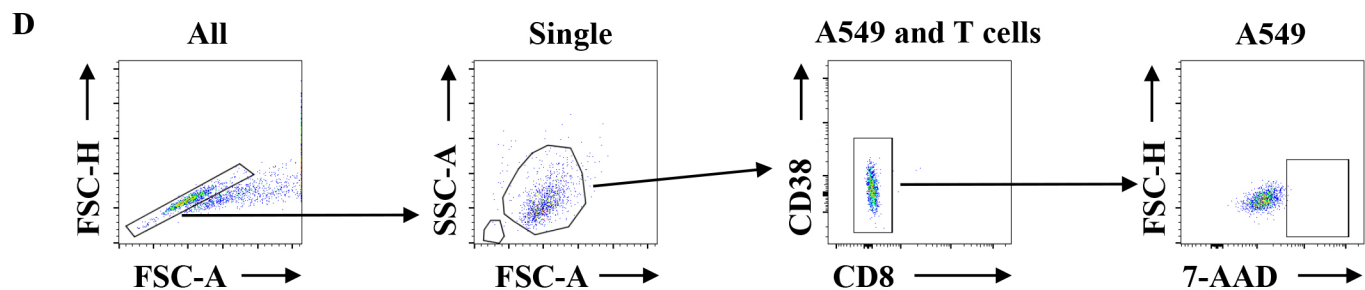

Supplement: Supplementary file 4 — Supplementary file4 (PDF 1043 kb) [file 262_2021_2949_MOESM4_ESM.pdf]
