# Supplementary material for: CD38 identifies pre-activated CD8+ T cells which can be reinvigorated by anti-PD-1 blockade in human lung cancer
Source: Cancer Immunol Immunother. 2021 May 2;70(12):3603–16. doi: 10.1007/s00262-021-02949-w (PMC8571140; doi:10.1007/s00262-021-02949-w)
Supplement: Supplementary file 5 — Supplementary file5 (PDF 113 kb) [file 262_2021_2949_MOESM5_ESM.pdf]

Supplementary Table 1. Characteristics of the Patients

Patient number	N=51
Age at enrollment—years	
Mean ±SD	64.27 ± 8.41
Median (range)	66 (41-83)
Sex-No. (%)	
Female	19 (37)
Male	32 (63)
Histologic diagnosis-No. (%)	
Adenocarcinoma	32 (63)
Squamous-cell carcinoma	17 (33)
Other	2 (4)
Clinical disease stage-No. (%)	
I	37 (72)
II	7 (14)
III	7 (14)
Smoking status-No. (%)	
Never	28 (55)
Former or current	21 (43)
